# Computational simulation of stent thrombosis induced by various degrees of stent malapposition

**DOI:** 10.3389/fbioe.2022.1062529

**Published:** 2022-11-14

**Authors:** Zhuoran Qu, Hongge Wei, Tianming Du, Aike Qiao

**Affiliations:** Faculty of Environment and Life, Beijing University of Technology, Beijing, China

**Keywords:** stent thrombosis, stent malapposition, dynamic thrombus formation, shear-induced thrombus aggregation, computational fluid dynamics, 3D continuum model

## Abstract

Percutaneous coronary intervention with stent implantation is one of the most commonly used approaches to treat coronary artery stenosis. Stent malapposition (SM) can increase the incidence of stent thrombosis, but the quantitative association between SM distance and stent thrombosis is poorly clarified. The objective of this study is to determine the biomechanical reaction mechanisms underlying stent thrombosis induced by SM and to quantify the effect of different SM severity grades on thrombosis. The thrombus simulation was performed in a continuous model based on the diffusion-convection response of blood substance transport. Simulated models included well-apposed stents and malapposed stents with various severities where the detachment distances ranged from 0 to 400 μm. The abnormal shear stress induced by SM was considered a critical contributor affecting stent thrombosis, which was dependent on changing SM distances in the simulation. The results illustrate that the proportion of thrombus volume was 1.88% at a SM distance of 75 μm (mild), 3.46% at 150 μm, and 3.93% at 400 μm (severe), but that a slight drop (3.18%) appeared at the detachment distance of 225 μm (intermediate). The results indicate that when the SM distance was less than 150 μm, the thrombus rose notably as the gap distance increased, whereas the progression of thrombogenicity weakened when it exceeded 150 μm. Therefore, more attention should be paid when SM is present at a gap distance of 150 μm. Moreover, when the SM length of stents are the same, thrombus tends to accumulate downstream towards the distal end of the stent as the SM distance increases.

## 1 Introduction

Every year, approximately 5 million percutaneous coronary interventions (PCIs) with stent implantation are performed worldwide to treat coronary artery disease (Torrado et al., 2018; Gori et al., 2019). The complications that arise from PCI are a major concern for a small but significant number of patients even if the complications occur at a relatively low incidence. Among the complications, the greatest concern is stent thrombosis (ST), which has a 5–45% mortality rate and a 15–20% recurrence rate at 5 years post-intervention. Stent malapposition (SM) has been most frequently found both in early and late ST cases (Koskinas et al., 2012; Mori et al., 2016; Adriaenssens et al., 2017; Im et al., 2019).

SM is one of the inadequate stent deployments and is defined as at least one stent strut being detached from the innermost layer of the vessel wall (Souteyrand et al., 2016; Hudrea et al., 2018). The detachment distance is calculated as the distance between the abluminal face of the strut and the vessel wall and usually ranges from 100 to 500 μm (Foin et al., 2014). Many factors contribute to SM, encompassing procedure-related factors (stent under-expansion, undersizing), plaque-related factors (thrombus dissolution, positive vessel remodeling), and stent-related factors (delayed endothelization, chronic stent recoil) (Taniwaki et al., 2016; Wei et al., 2021). Well-apposed struts can be classified as embedded struts or protruding struts (Giglioli et al., 2020). Embedded struts are buried into the vessel wall and are regarded as “stable.” Although protruding struts are covered by neointima, they represent a foreign body interrupting blood flow near the vessel wall. Therefore, protruding struts and malapposed struts are both considered “vulnerable.”

To investigate the effect of protruding stents on thrombus formation, an *in vitro* experiment using a step model found that thrombus deposit increased when the protruding part of the struts became larger (Corbett et al., 2010). In other *in vitro* experiments with stents employments, the protruding strut produced less thrombus than in a malapposed situation (Ng et al., 2022) and for the malapposed stents thrombogenicity of the stent rose in tandem with larger SM distances (Foin et al., 2017; Perry-Nguyen et al., 2020).

The hemodynamic mechanism underlying this phenomenon has been studied using computational fluid dynamics (CFD). Some 2D and 3D CFD models have been used to understand how SM distance affects blood flow and thrombogenicity. The protruding and malapposed stent struts disturbed the blood flow and created micro-recirculation, which reduced the flow rate and shear stress, leading to higher blood viscosity and promoting the stagnation of platelets and coagulation factors (Poon et al., 2018). This led to the assumption that SM might be a trigger for the thromboembolic events (De Santis et al., 2013; Rikhtegar et al., 2013). In addition, it has been found that the size and position of the micro-recirculation are related to the SM distance (Chen et al., 2017) and that disturbances in the flow tend to worsen with increasing detachment distance, resulting in enhanced thrombogenicity (Foin et al., 2014; Hudrea et al., 2018). Interestingly, some studies have argued that SM distances might not necessarily produce a greater hemodynamic disturbance when compared to smaller SM distances (Chesnutt and Han, 2016; Gori et al., 2019). According to a patient-specific coronary artery model, the area of adverse wall shear stress in cases of SM is smaller than well-apposed cases (Wei et al., 2021). Other researchers have utilized SM models with different detachment distances to simulate the aggregation of platelets and found that the relationship between detachment distances and thrombogenicity is not always positively correlated (Chesnutt and Han, 2016).

In the aforementioned studies, the correlation between SM distance and ST was generally inferred by comparing the results of CFD simulations and clinical observations or *in vitro* experiments. Most studies were based on qualitative analysis without the process of thrombus formation, which makes it difficult to investigate differences in the severity of thrombus induced by various degrees of SM. Most studies have focused on the effect of SM on flow stagnation as opposed to the biomechanical association between blood flow and thrombus growth. As ST is correlated with platelet activation and coagulation, it is necessary to include blood substances in fluid simulations (Zhang et al., 2022).

Therefore, it is necessary to simulate the dynamic thrombus formation in a SM model in order to precisely clarify variations in ST attributed to different SM distances. The objective of this study is to delineate the biomechanical reaction mechanisms underlying ST induced by malapposition and to quantify the effect of various malapposition severities on thrombosis. We simulated deposition of thrombus in 3D stent-vessel models with different levels of SM and compared the data with previous *in vitro* experiments on ST to validate our simulation results.

## 2 Methodology

Idealized well-opposed and malapposed models were developed. Blood flow was simulated using CFD and was integrated with the continuous thrombus calculation model. Regions with high probability of thrombus formation were determined by calculating local wall shear stress, residence time, and shear rate. In this section, the details of the thrombus model and the dynamic calculation procedures are described.

### 2.1 3D SM models

**FIGURE 1:**
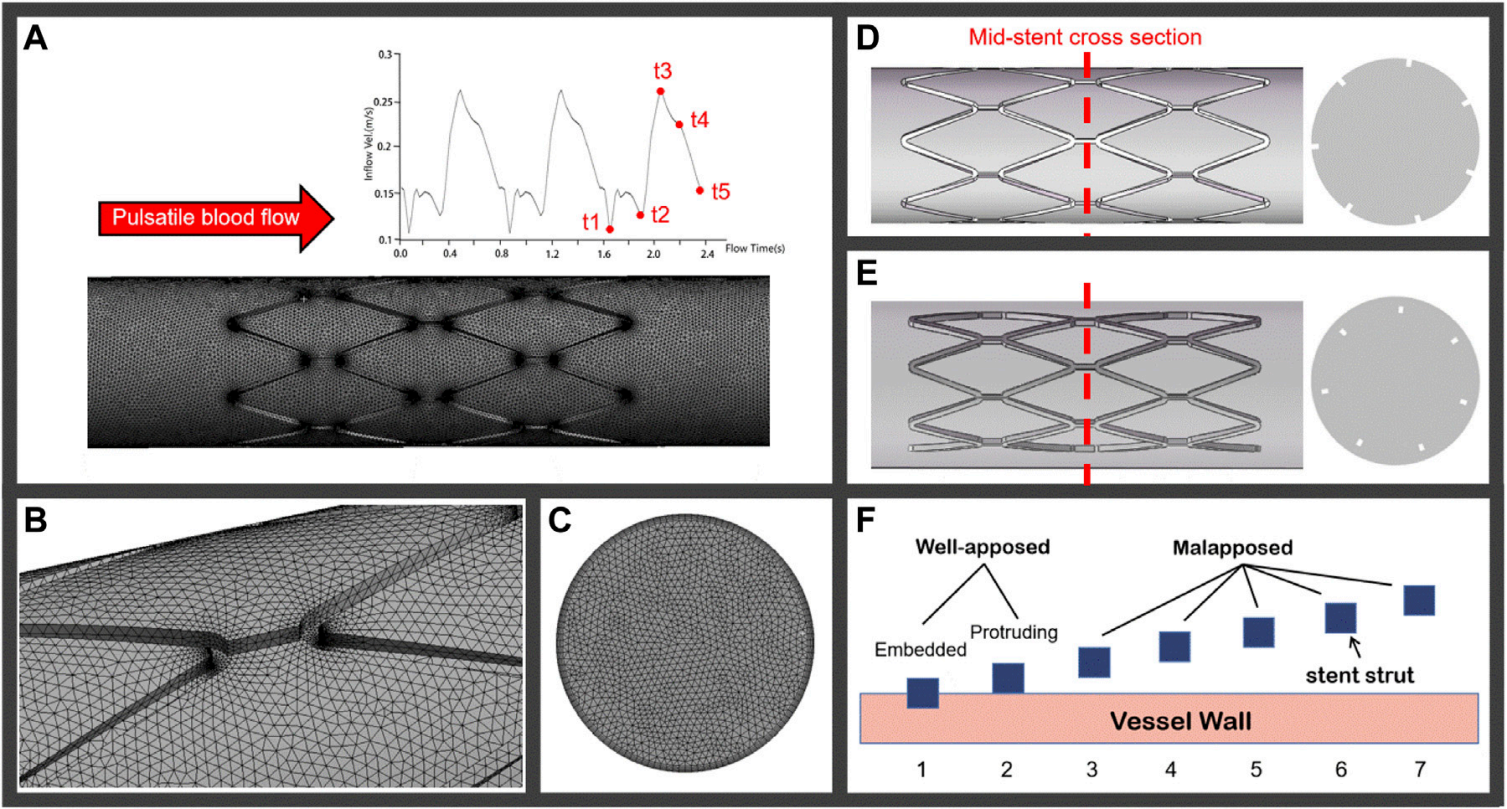
**(A)** Pulsatile coronary blood flow is applied at the inlet of the computational domain for CFD analyses and a meshed computational domain inside an idealized straight vessel; **(B)** tetrahedron elements on the lumen surface; **(C)** increase in mesh density near the inflation layers; **(D)** and **(E)** represent the well-apposed and malapposed idealized stent-vessel model and their cross-sections, respectively; **(F)** stent struts in seven cases categorized as well-apposed and malapposed, the struts in case 1 are simulated as embedded stents and the detachment distances increase from case 2 to case 7.

According to the European Association of Percutaneous Cardiovascular Interventions (EAPCI), when more than half of the stent strut thickness is buried into the vessel wall, this is defined as an embedded strut, while a protruding strut refers to a strut where less than 50% of the strut thickness is buried into the vessel wall. A stent with a SM distance of more than 400 μm is considered a severe SM (Foin et al., 2014; Naganuma, 2020). Based on the struts scenarios of several types of stents, vessels in this study were assumed as a rigid circular pipe with a diameter of 3.5 mm. The stent model employed was generated as a circular equal-diameter stent with a strut thickness of 0.15 mm. Cases 1 and 2 were modeled as embedded struts and protruding struts, respectively, under the well-apposed category, as shown in Figure 1F. The detachment distances in malapposed models, ranging from 75 μm (Case 3) and 225 μm (Case 5) to 400 μm (Case 7), were classified as mild, intermediate, and severe SM, respectively. Figures 1D,E illustrated the stent-vessel model of case 2 and case 5, respectively. Some researchers have simplified the stent into circular rings to mimic SM (Poon et al., 2018) and only investigated blood flow patterns located in a single strut. The length of the stent extended to 8 mm in our models, enabling us to investigate longitudinal hemodynamic changes and thrombus growth. All stent and vessel models were constructed using the 3D computer aided design software SOLIDWORKS 2016.

### 2.2 Numerical simulation approaches

#### 2.2.1 Thrombus formation

**FIGURE 2:**
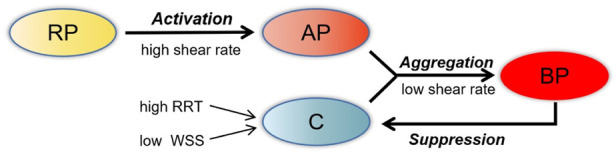
Schematic depiction of the thrombosis model. RP, resting platelets; AP, activated platelets; C, coagulation; BP, bounding platelets; RRT, relative residence time; WSS, wall shear stress.

The thrombus model used in this simulation was developed by Menichini and Xu (2016). Low shear rate tends to induce the deposition of platelets and coagulation factors. Hence, shear rate, relative residence time (RRT), and time-averaged wall shear stress (TAWSS) were adapted as hemodynamic parameters to characterize the distribution of stagnancy and shear rate level in the flow. Thrombus formation was simulated by solving the advection–diffusion equation, which describes the transport process of coagulation (C), resting platelets (RP) and activated platelets (AP), shear rate, and RRT with their switching functions determining accumulation of bound platelets (BP). Thrombus is represented by BP concentration. In the model, C represents a series of coagulation factors that participate in the thrombosis reaction, although its concentration does not directly represent a specific coagulation factor. With this thrombus simulation framework, the formation of C was promoted by low TAWSS (<0.2 Pa) and high RRT (>0.9). In the local hemodynamic environment, AP and high concentration of C promoted thrombus formation, while local high concentrations of BP on the vessel wall depressed the formation of C, as in Figure 2. The governing equations of user-defined scalars were provided in the study of Menichini and Xu (2016). In this study, a number of important changes were made to this thrombus model, which are illustrated in the following paragraphs.

The present thrombus model only considered low shear stress as a reaction term to control the flux input of coagulation and as a factor to increase stagnancy. It is commonly known that high shear stress induced by malapposed struts can activate platelets, which contributes to thrombus aggregation (Chesnutt and Han, 2016). Hence, the reaction source term Si of AP and RP was altered to a sum of two terms as follows (Eq. 1):

$$S_{i}=K_{1}\left[AP\right]\left[RP\right]+K_{2}\left[RP\right]\dot{\gamma }\mu$$, (1)

where the first term on the right represents the activation effect of RP by AP, the second term on the right represents the activation by high shear stress, μ is blood viscosity, $\dot{\gamma }$ is the time average shear rate, and $K_{1}$ and $K_{2}$ equal 0.5 $s^{-1}$ .

For the initial CFD simulation, blood was treated as shear-thinning fluid as described by the viscosity model developed by Quemada (Marcinkowska-Gapińska et al., 2007), which was adapted to incorporate the reported hematocrit of 35%. In order to simulate the resistance to blood flow after thrombus development, local elements that corresponded to “thrombus formation” were defined as viscoelastic, which was determined using the viscosity function related to flow shear rate and BP (Eq. 2):

$$\mu =\left\{\mu_{0}+\mu_{\infty }{\left[1+{\left(\alpha \dot{\gamma }\right)}^{2}\right]}^{n}\right\}\left(1+100\frac{{BP}^{2}}{{BP}^{2}+{BP}_{t}^{2}}\right),$$ (2)

where $\mu_{0}$ = 3.45cp and $\mu_{\infty }$ = 56cp, representing blood viscosity at the infinite shear rate and zero shear rate, respectively, α = 3.313 s represents the relaxation time, and *n* = −0.3216 (Qian et al., 2020). ${BP}_{t}$ was set at 20 nmol and the higher the BP concentration, the greater the viscosity of local elements in order to imitate the resistance to blood flow.

#### 2.2.2 Calculation procedures

Numerical simulations of blood flow in the stented vessel were carried out by solving the Naiver–Stokes equations and transport equations using the volume-based algorithm Fluent (v19.0, ANSYS) with user-defined functions (UDF) and user-defined scalar (UDS). Blood was assumed to be an incompressible non-Newtonian fluid with a constant density of 1050 kg/m^3^. In each simulation, blood flow CFD was first run over three cardiac cycles, with a cardiac cycle of 0.8 s and time-step increment of 0.01 s. The third cycle was used to calculate RRT, TAWSS, and shear rate over one cycle. The thrombus model was subsequently introduced to simulate formation of thrombus for 1 s. The aforementioned calculation procedure was repeated over five rounds in all the models and the thrombus was accumulated for 5 s in total. After each formation of thrombus, the renewed viscosity coefficient was adapted in the next round of simulation to imitate the resistance present during thrombus growth. The higher the concentration of BP in a local area, the greater the obstruction of blood flow.

The fluid domain was discretized into tetrahedral elements, as in Figure 1B. Figure 1C shows the meshes on the cross section where the mesh density increases near the inflation layers. The computational domain had approximately 4,446,653 elements, and the tetrahedral mesh had a maximum face size of 0.08 mm. Finer meshes with 5,462,027 elements were created to assess mesh sensitivity. Differences in TAWSS peak values between meshes were less than 3%, indicating that mesh independence had been achieved.

#### 2.2.3 Boundary conditions

A transient velocity waveform that imitated the coronary blood flow was applied at the model inlet, as shown in Figure 1A, while the outlet pressure was set as constant zero. A rigid wall was imposed on the fluid domain with a no-slip condition. The coagulation factors and platelets boundary conditions used were similar to those previously described by Menichini and Xu (2016).

## 3 Results

### 3.1 Blood flow characteristics

**FIGURE 3:**
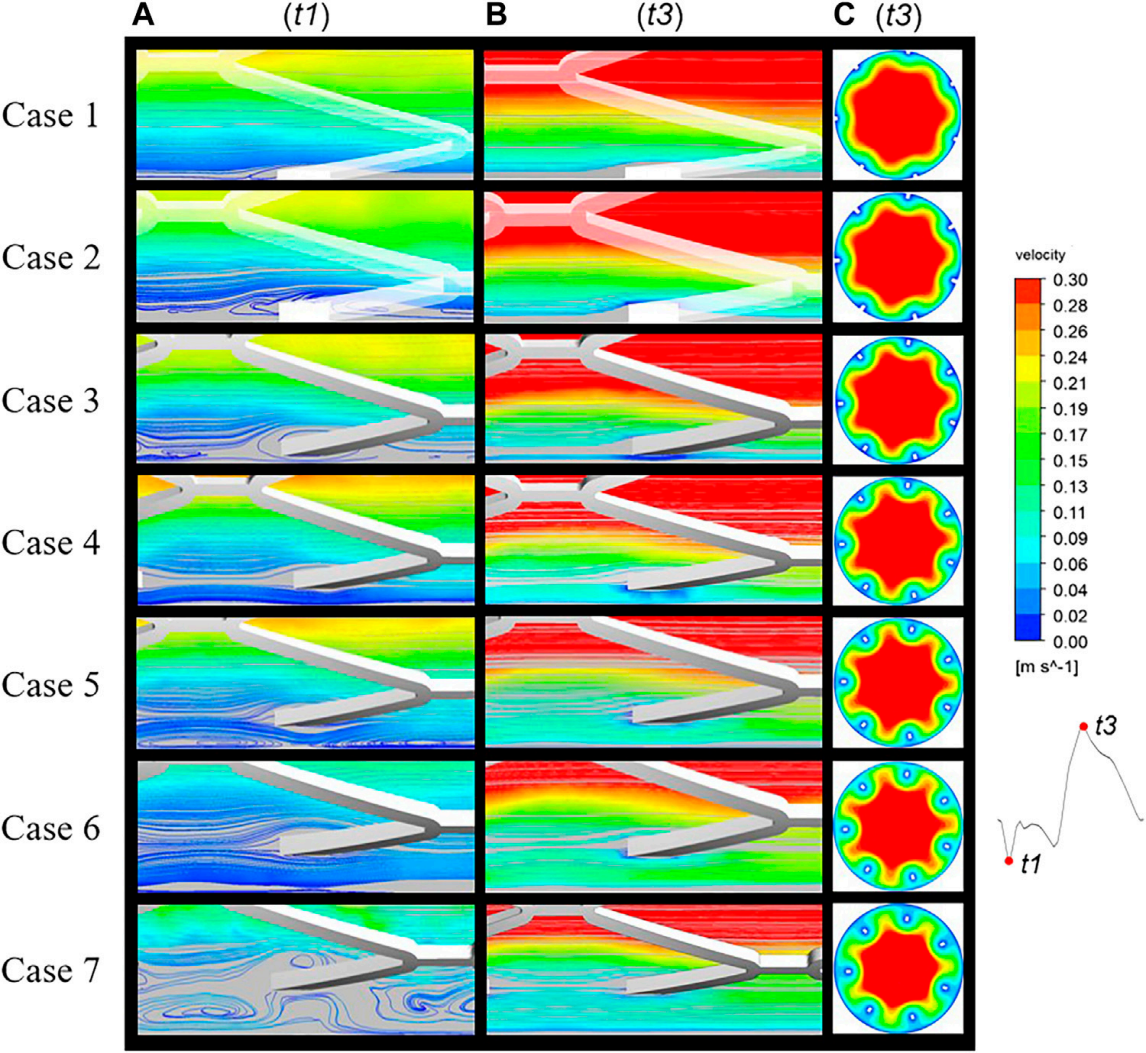
Blood flow streamlines at two time points of the cardiac cycle: t1, nadir diastole; t3, peak systole. The flow near the stents was evidently more disturbed at **(A)** (t1) than **(B)** (t3). **(C)** Mid-stent cross-sections of all cases with the regions of low velocity near the struts having migrated from the vessel wall to the interior.

Velocity streamlines were assessed in the longitudinal section of cases 1 to 7 at two time points, t1 and t3, representing inlet velocities at nadir diastole and peak systole, respectively (Figures 3A,B). In case 1, streamlines are smooth over the embedded struts at both time points. However, in case 2, disturbed flow was found near protruding struts with the appearance of a small micro-recirculation on the backward step of the struts, as shown in Figure 3A. In case 3, the recirculation region is larger and surrounds the malapposed struts. With an increasing detachment distance, the malapposed struts separate the blood flow but without micro-recirculation, as seen in cases 4 and 6. In case 5, a slight micro-recirculation is present adjacent to the vessel wall rather than the stent struts. In case 7, two recirculation regions located both proximal and distal to the struts joined to form a large region of disturbed flow enveloping the stent. The distribution of flow velocity in mid-stent cross-sections of the stented area at time point t3 is shown in Figure 3C. Figure 1D shows the location of the cross-sections. A low-velocity region is seen around the stent struts in malapposed cases and moves inwards with increasing SM distances, which is the result of the viscous effect of blood between the stent struts and vessel wall. Overall, fluid perturbations in all stents are greater under the minimum velocity compared to the maximum velocity (Figure 3B). The most pronounced disturbances are observed in the maximum detachment distance, as in case 7. Flow field patterns were significantly affected by various malapposition degrees.

### 3.2 Wall flux properties and RRT distributions

**FIGURE 4:**
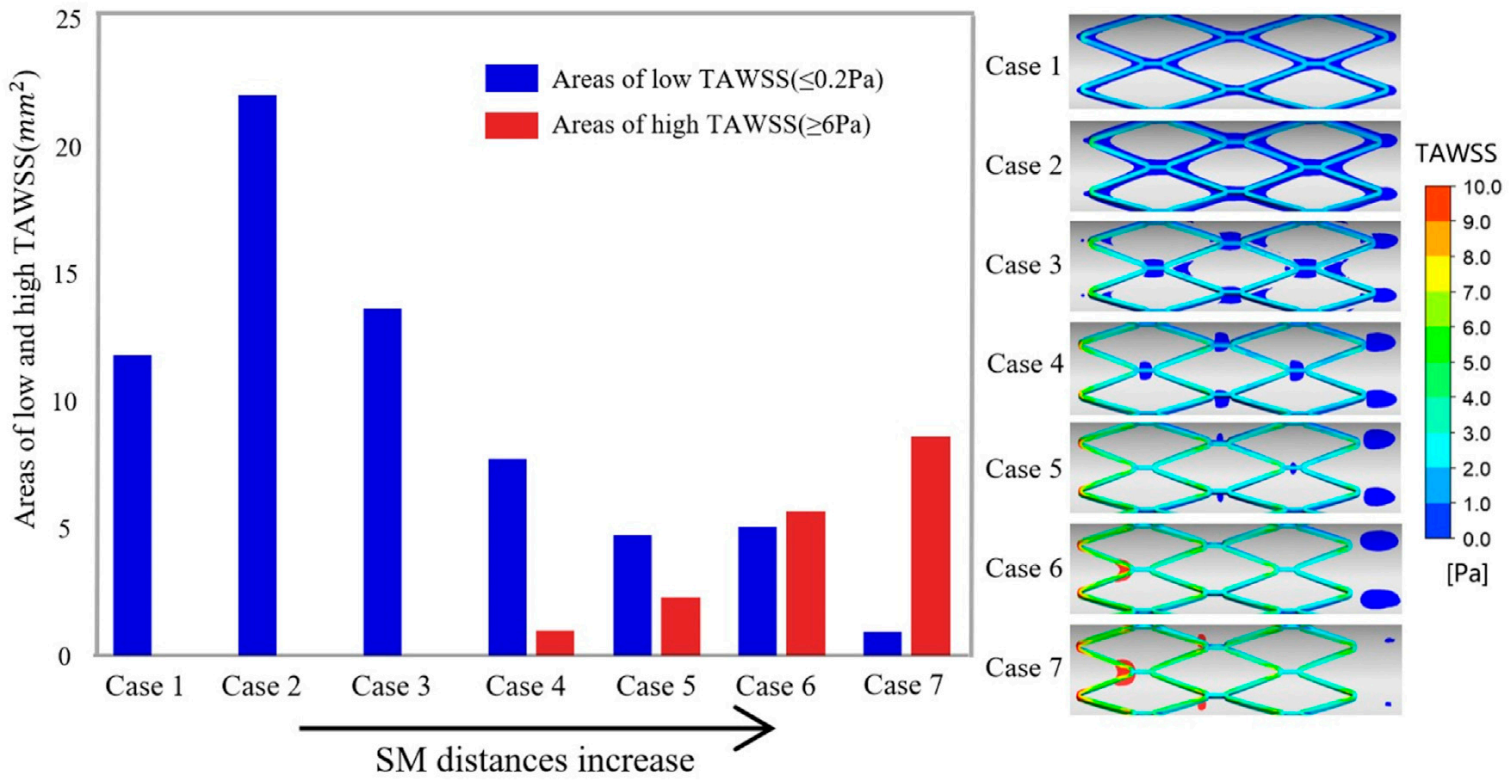
Right panel illustrates the complete TAWSS contours on the stent surface and the region of low TAWSS (≤0.2 Pa) and high TAWSS (≥6 Pa) on the vessel wall are colored blue and red, respectively. The histogram shows the area of adverse TAWSS from case 1 to case 7 with the increasing SM distance. The areas correspond to the highlighted region of TAWSS on the vessel wall. TAWSS, time-averaged wall shear stress.

The histogram in Figure 4 presents the areas of low and high TAWSS on the vessel wall for all cases, which are arranged in order of increasing SM distance. In the thrombosis model, coagulation factors were produced within the area of low TAWSS (≤0.2 Pa) and platelets were activated upon high shear stress (≥6 Pa). In TAWSS contours (Figure 4), regions subjected to low and high TAWSS were isolated and colored blue and red, respectively, and the full range of contour colors is displayed on the stent surfaces. For apposed stents, in case 1 and case 2, low TAWSS surrounded the stent struts and the area within a protruding stent was twice that of an embedded stent. For cases 3 and 4, low TAWSS was localized downstream of the stent connectors. As the detachment distances increased, low TAWSS was gradually concentrated at the distal ends of the vessel wall. The area of low TAWSS exhibited continuous reduction from cases 2 to 7, while high TAWSS emerged at the proximal region in case 4 and increased with the enhancing severity of malapposition. The area of high TAWSS is about nine times larger than that of low TAWSS in severe SM, as reflected in case 7. In this study, low TAWSS tended to occur in the well-apposed and intermediate SM cases where the detachment distance is relatively small; the values of TAWSS on the entire stent surface increased from case 1 to case 7.

**FIGURE 5:**
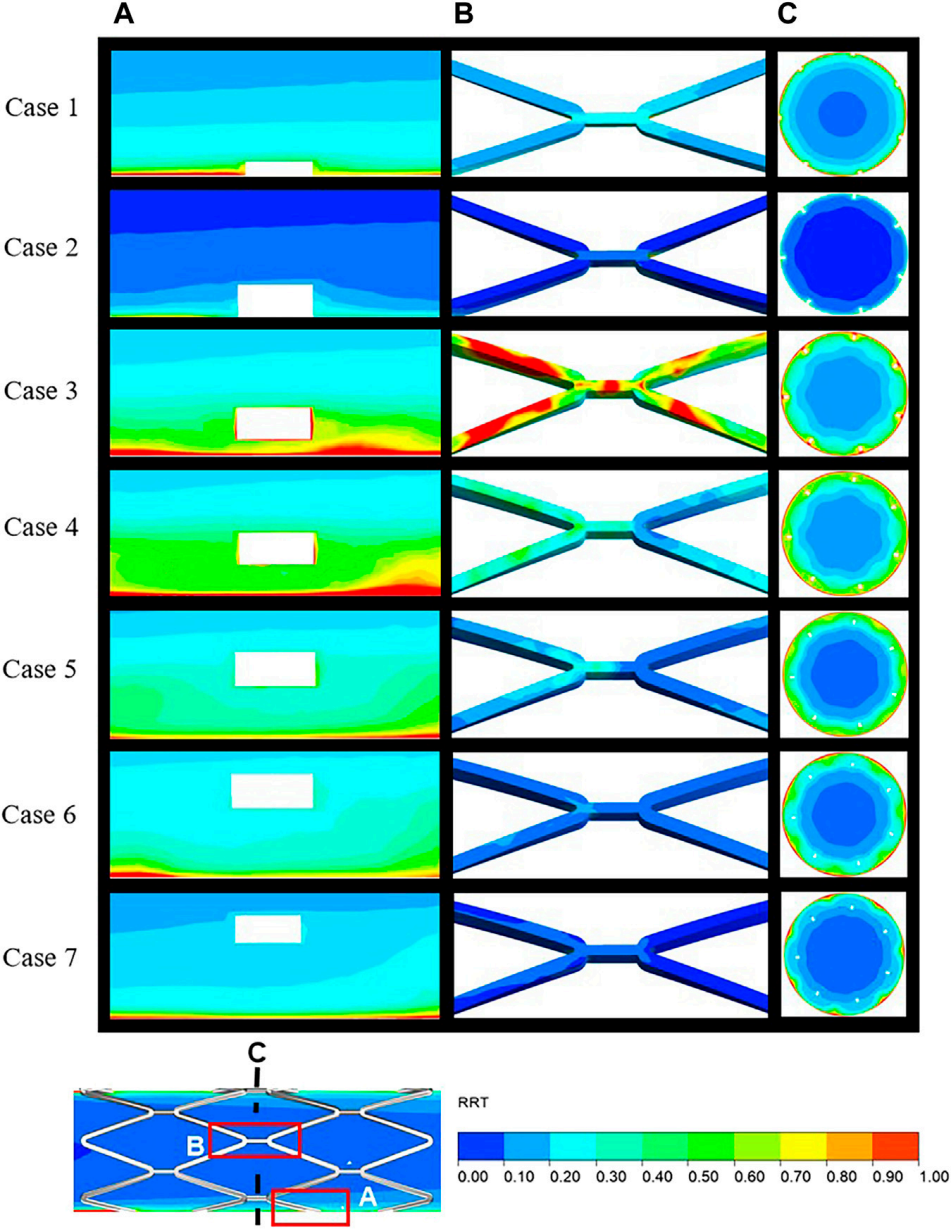
Distribution of RRT over the local area in the longitudinal sections **(A)**, stent surface **(B)**, and mid-stent cross-sections **(C)** (locations are illustrated in Figure 6). RRT, relative residence time.

The distribution of RRT in longitudinal sections, stent surfaces, and mid-stent cross-sections is shown in Figure 5. In Figure 5A, regions with high RRT gradually moved downstream along the boundaries with the increasing SM distances and a similar pattern was observed in the movement of low TAWSS on the vessel wall. Figure 5B shows the stent struts at the middle position of the stent segment. The smallest SM distance of case 3 in malapposed cases had the highest RRT on stent surfaces, with the RRT on the stent surfaces gradually decreasing from case 3 to case 7. High RRT implies an increase in concentration of coagulation factors that is linked to thrombus formation. For the cross-sections in Figure 5C, the region with high RRT was consistent with the region of low flow velocity for case 1 to case 4 and was mainly distributed around the stent struts and vessel wall. For cases 5 to 7, regions with high RRT moved from stent struts to the vessel wall and were eventually distributed between the adjacent struts, while low RRT was distributed around the struts. The combination of high RRT and low TAWSS might increase the likelihood of platelet deposition surrounding the malapposed struts.

### 3.3 Thrombus formation

**FIGURE 6:**
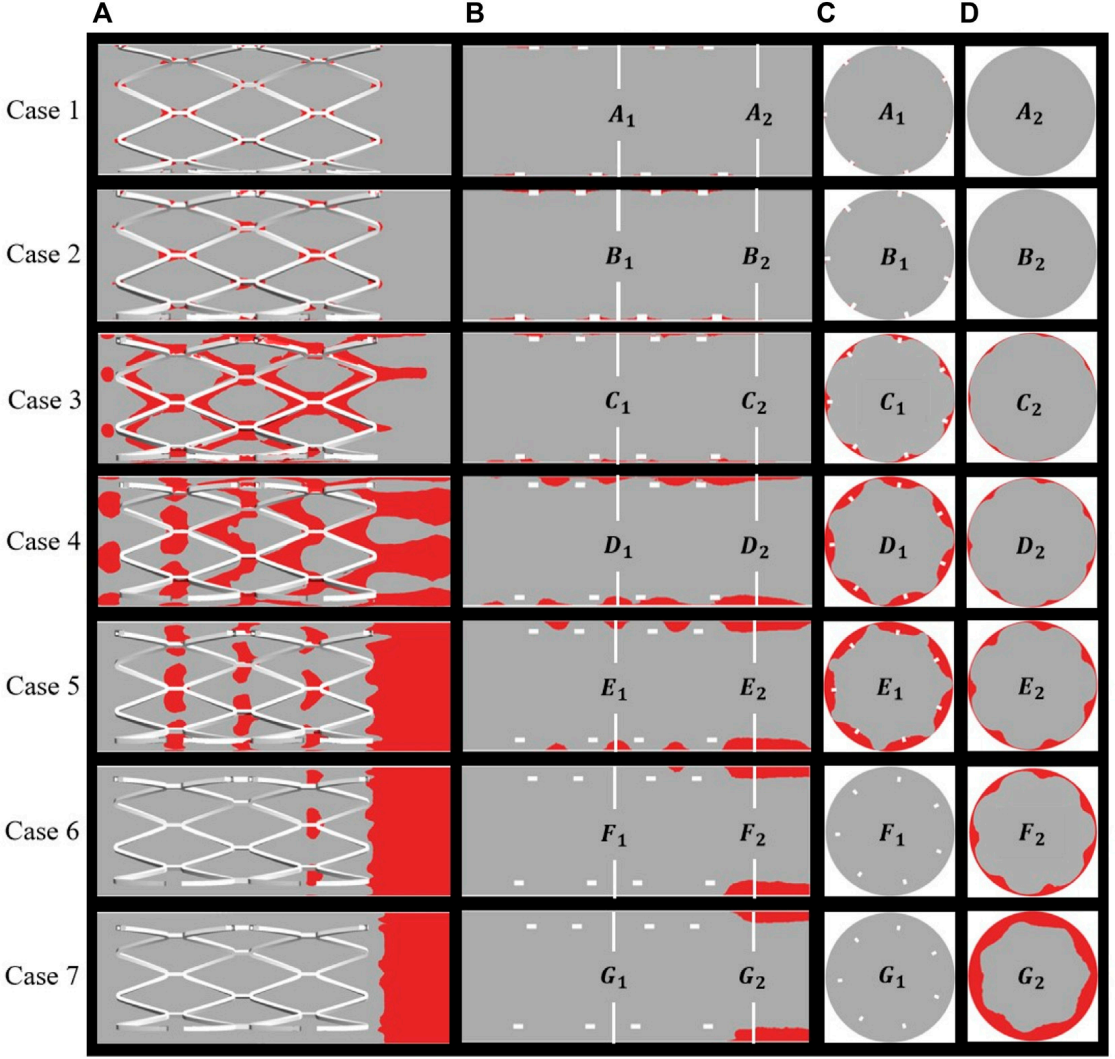
Distribution of thrombus at the end of simulation: **(A)** thrombus on the overall perspective; **(B)** thrombus on the longitudinal sections and locations of two cross-sections; **(C)** middle location of the stents; **(D)** distal end of the stents.

BP is regarded as a sign of thrombosis and, in this study, the threshold of BP was fixed at 8 nmol/L. Figure 6 shows the distribution of thrombus for all cases as calculated from the hemodynamic data. Thrombus on the stent surface and vessel wall is shown in Figure 6A and thrombus on the longitudinal sections, mid-stent cross-sections, and distal cross-sections are depicted in Figures 6B–D, respectively. For well-apposed cases 1 and 2 in Figure 6A, only a small degree of thrombus was observed around the stent connectors and thrombus covered a small area on the longitudinal sections and cross-sections (Figures 6B,C). In case 3, thrombus was observed on every strut of the stent, and the cross-section showed that the thrombus grew inward and filled the space between the vessel wall and stent struts and a small amount of thrombus shows on the distal end of the stent. With increasing SM distance, thrombus tends to accumulate on the downstream side of stent segment for case 4 to case 7. Decreasing RRT could be responsible for the reduction in thrombus at the middle position of the stents and the pattern of thrombus movement was in agreement with the distribution of low TAWSS (Figure 4). In case 4, thrombus tended to deposit downstream of the stent strut and covered the whole cross-section of the struts. For case 5 in Figure 6A, the region on the vessel wall covered by thrombus is smaller, while the extent of thrombosis as viewed from both longitudinal sections and cross-sections remained mostly unchanged compared to case 4. As in Figure 6D, for cases 6 and 7, thrombus was only generated at the distal half rather than the middle of the stent. SM distances not only affect the thrombus area on cross-sections bus also the location of thrombus in the longitudinal direction.

**FIGURE 7:**
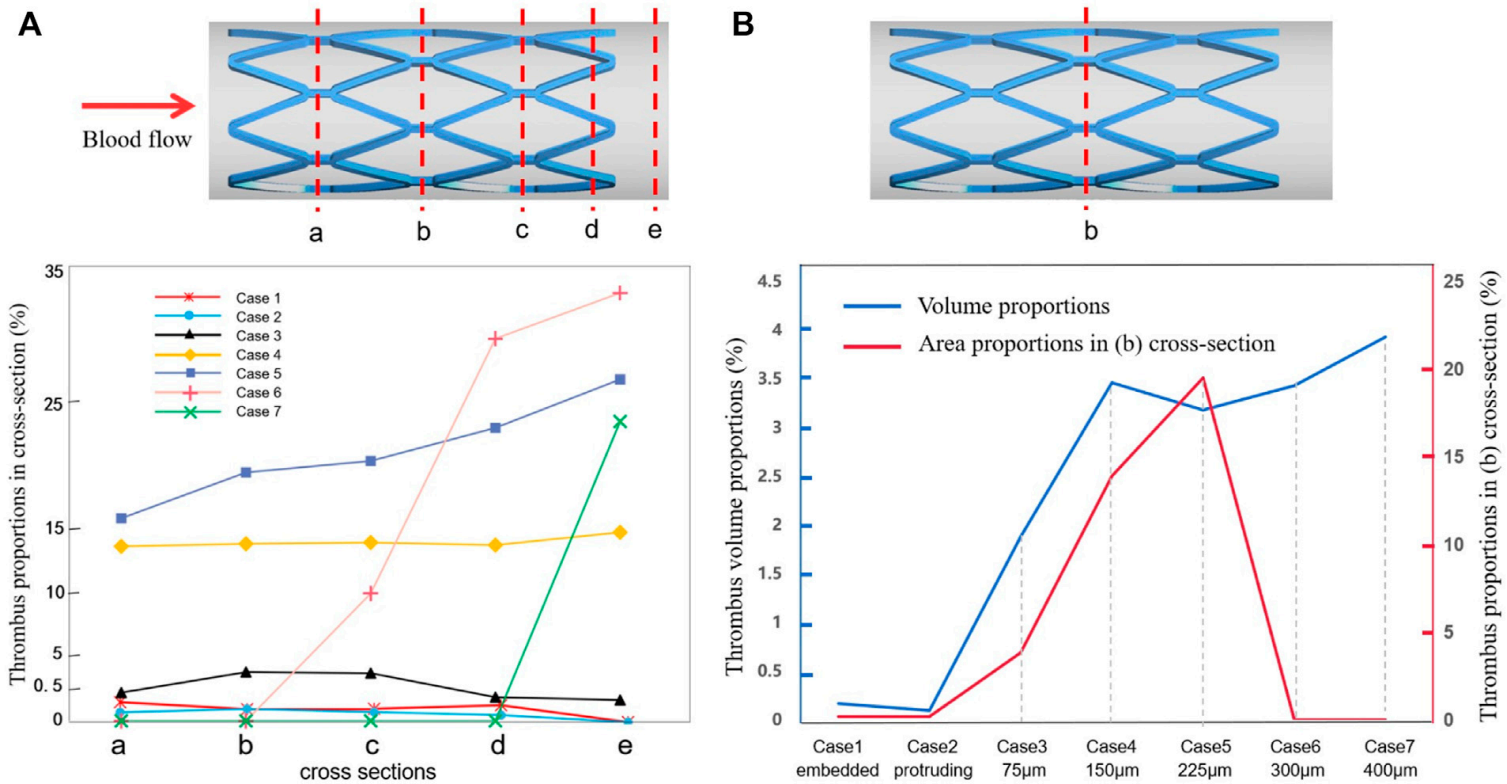
Thrombus accumulation amount at the end of simulation: **(A)** thrombus proportions in five cross-sections (a–e) of all cases; **(B)** thrombus area proportions in the mid-stent cross-section (b) and thrombus volume proportions in the fluid domain.

Figure 7A shows the area percentage of thrombus on the five cross-sections (a–e) for all cases. For case 1 to case 4, there was little difference in the distribution of thrombus from upstream to downstream of the stent, where the larger the SM distance, the higher the thrombus amount. For cases 5 to 7, a higher amount of thrombus tended to accumulate on the distal end of the stent rather than being uniformly distributed, as can be seen from the slope of the graph (the longer the distance, the more significant the trend). Figure 7B shows the thrombus area proportions in mid-stent cross-section (b) and the thrombus volume proportions with increasing degree of SM. The thrombus volume increased greatly from case 2 to case 4; case 4 had severe thrombosis with the proportion of thrombus volume at 3.46%, which is 35 times that measured for case 2. The proportion of thrombus volume showed a slight downward drop in case 5, with a 3.18% thrombus volume. From case 4 to 7, the severity of the thrombus continued to grow but was less significant; it increased by 14% from case 4 to case 7. The thrombus area proportions in cross-section (b) keep rising from case 1 to case 5, then dropping to zero for cases 6 and 7, most likely because the thrombus deposit moves downstream to the distal end. Overall, there was a rising trend of thrombus presentation in the entire stented segment; in severe malapposed cases, thrombus proportions still increased but were mostly concentrated on a location downstream of the stented segment, which is technically a non-stented area.

### 3.4 Relationship between viscosity and thrombus

**FIGURE 8:**
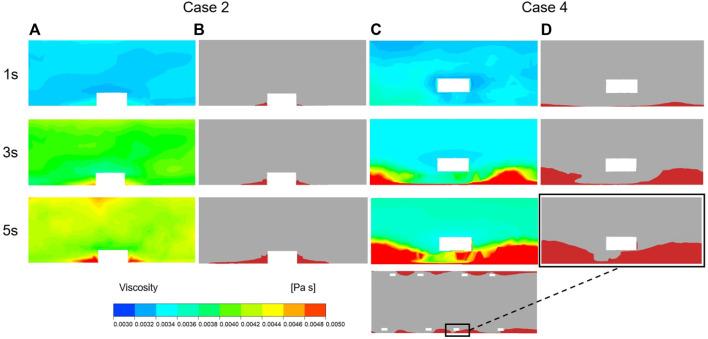
Viscosity and thrombus distribution on the longitudinal sections of case 2 and case 4 at three time points of simulation. **(A)** Viscosity distribution of Case 2 after 1 s, 3 s and 5 s of thrombus calculation. **(B)** Thrombus distribution of Case 2 after 1 s, 3 s and 5 s of thrombus calculation. **(C)** Viscosity distribution of Case 4 after 1 s, 3 s and 5 s of thrombus calculation. **(D)** Thrombus distribution of Case 4 after 1 s, 3 s and 5 s of thrombus calculation.

The method for simulating resistance in blood flow of the thrombus has been explained previously. According to Eq. 2, the viscosity of blood flow was associated with shear rate and the concentration of BP, where the local elements presented more viscoelasticity with higher BP values. Figure 8 illustrates the variation in viscosity and growth of thrombus for case 2 and case 4 over the accumulated simulation time. Prior to calculating the time of initial thrombus formation, blood was treated as shear-thinning fluid. As shown in Figure 8A, initially, higher viscosity appeared on the corners of both sides of the protruding struts. Meanwhile, for case 4, lower viscosity was noted at the location around the struts and near the vessel wall and a thin layer of higher viscosity was found closer to the malapposed struts. The viscosity contours at 3 s showed that viscosity increased dramatically with high BP values (Figures 8A,C). In the next simulation, viscosity distribution and hemodynamic characteristics affected thrombus accumulation, as seen in Figures 8B,D, where regions of high viscosity were consistent with the formation of thrombus at 3 s and 5 s. For case 2, thrombus was localized to both sides of the protruding struts. On the other hand, in case 4, thrombus presented on the vessel wall first and then grew inward between the adjacent struts, eventually attaching the malapposed struts.

## 4 Discussion

In this study, we simulated the formation of thrombosis based on the diffusion-convection response of blood substance transport. We investigated variation in the amount of thrombus formed under the different degrees of SM and discussed the association between ST and hemodynamic patterns. The formation of BP is directly influenced by the transport of C and AP, whose values depend on the distribution of shear stress and residence time. Higher residence time and low shear stress both directly contributed to a higher thrombosis value. According to a reported hemodynamic analysis based on patient-specific CT scans, areas with TAWSS less than 0.2 Pa corresponded to the location of thrombosis (Menichini et al., 2016), where coagulator input occurred in our simulation. A lower or higher threshold would cause slower thrombus growth or overestimate the amount of thrombus formed. To a certain extent, high shear stress could indirectly lead to thrombus formation by activating platelets. For example, shear stress of more than 6 Pa has been found to be more likely to induce platelet activation compared to normal shear stress (Chesnutt and Han, 2016). We illustrated the distribution of high and low shear stress in Figure 4 to indicate the potential regions of thrombus formation under different SM distances.

It has been suggested that an increase in local blood viscosity may be a significant contributor to ST near malapposed struts (Poon et al., 2018). Thus, it is necessary to consider blood as a shear-thinning fluid with a dynamic viscosity when performing CFD. According to Anand et al. (2003), clot viscosity is much greater than blood viscosity; therefore, in our study, thrombus was included as one of the factors affecting viscosity. Viscosity acted as a marker of the changing hemodynamic characteristics influenced by growing thrombus. The greater the concentration of BP in a specific position, the greater the obstruction in fluid flow. Together, these parameters enabled the imitation of blood resistance caused by thrombus.

Some studies have proposed that a malapposed stent usually has a smaller area of adverse wall shear stress (WSS) than a well-apposed stent (Wei et al., 2021). Although our study observed that the region with low WSS decreased as the degree of SM increased, the area with high WSS increased with the increasing SM distance, as noted in cases 4 to 7. In the preliminary simulation, when we did not take into account high shear-induced platelet activation, due to the decreasing region of low WSS, we observe that the amount of thrombus continued to decline as the SM distance increased, which is contradictory to most clinical observations and *in vitro* experimental results. Taking into consideration the shear-induced platelet activation process, some modifications were included when simulating thrombus formation. In our study, low shear stress promotedthe accumulation of platelets and stagnation of blood flow, while high shear stress activated RP in the local area. We found that the area of thrombus generally increased with the increasing degree of SM but the thrombus volume of a protruding stent was not larger than that of an embedded stent and a slight decrease was noted for case 5 (225 μm). The trend in thrombus development was much more remarkable within a gap distance of 150 μm compared to distances that exceeded 150 μm. Thus, more attention should be paid to situations where the SM gap distance is of 150 μm, which is equal to the thickness of the stents in our models.

Previously, it has been shown that strut size could affect the local hemodynamic environment whereby thinner struts tend to cause smaller turbulence to the flow and lead to less thrombus (Beier et al., 2016). Chesnutt and Han (2016) simulated SM with a stent thickness twice as small as ours but the trend in thrombus variation was similar. A drop in the degree of thrombus formed appeared at the intermediate SM distance. The largest detachment distance (200 μm) produced more thrombi than the smallest detachment distance (10 μm) but less thrombus than an intermediate detachment distance (25 μm). This similarity in thrombus was attributed to the range of SM distances and the strut size in their simulation being proportionally smaller. Therefore, we assume that both detachment distance and stent thickness could influence ST. The amount of thrombus decreased at intermediate SM because the platelets did not experience more adverse shear stress (both high and low shear stress) around struts with the intermediate detachment distances (225 μm). This suggests that a large detachment distance does not necessarily reflect enhanced thrombogenicity. Even though larger SM distances may not produce a higher degree of thrombus, intermediate or severe SM should be avoided at the clinical level as it would otherwise induce stenosis and delayed neointimal healing (Wei et al., 2021).

A previous *in vitro* experiment simulated thrombus deposition using an artificial step model (Corbett et al., 2010). The investigators found that more thrombus was formed in parallel with the higher steps and this aligns with the data for the embedded case and protruding case in our study where thrombus formation within the cross-sections in case 2 was slightly more than case 1 because of the larger recirculation created by larger steps. The *in vitro* experiment also identified more thrombi on the backward step rather than the forward. In our study, more thrombi were deposited on the downstream side of the stent struts in cases 3 and 4 where the thrombi tended to accumulate downstream to the distal end of the entire stented segment as SM distances increased. Though the steps and malapposed stents are not identical, a foreign stent in the flow creates a larger recirculation vortex on the backward side, thus leading to more flow stagnation. As the detachment distance increases, the region of flow disturbance becomes larger and causes more thrombi deposit downstream. Figure 4 illustrates the same trend for low TAWSS.

**FIGURE 9:**
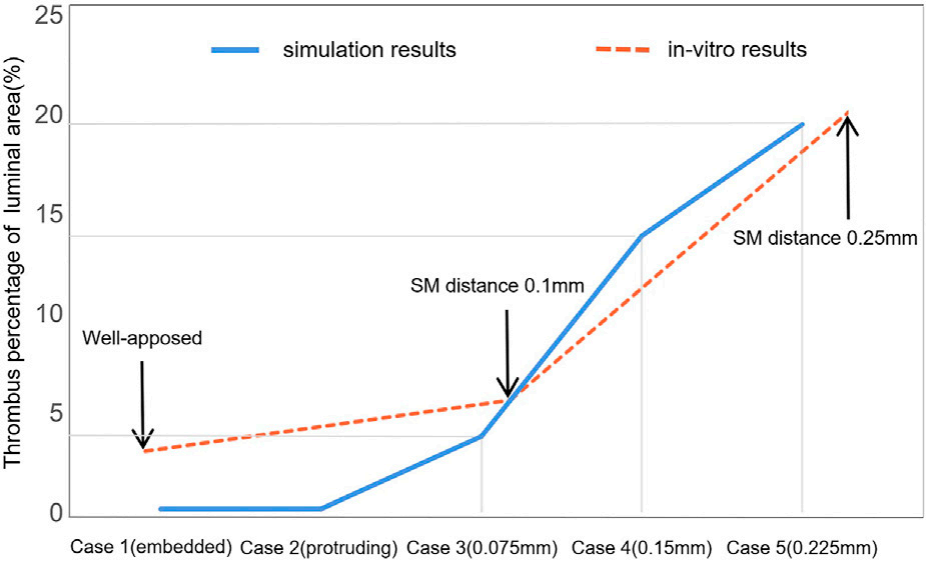
Comparison of thrombus amounts between numerical simulation and previous *in vitro* experiments. This illustrates the thrombus proportions of the luminal area with the increasing detachment distance. The two sets of results share the same axial coordinate.

Another previous *in vitro* experiment investigated ST with an incomplete stent apposition (Perry-Nguyen et al., 2020). The thrombus amount was indicated by the stent occlusion of the luminal area and the results of our study and the previous *in vitro* experiment were compared and are displayed in Figure 9. The experimental results were acquired by optical coherence tomography after 15 min perfusion through stents with varying degrees of malapposition relative to the lumen wall. Thrombus burden increased tremendously from well-apposed (0.00 mm) to malapposed (0.25 mm) from the lumen wall; a similar trend can be found in case 1 to case 5. The largest SM distance in our simulation was 0.4 mm in case 7 with a thrombus volume proportion of 3.93% and deposited on the distal non-stented location. With the *in vitro* experiment, stent thrombus almost completely occluded the tube at a SM distance of 0.5 mm; thus, a distance of >500 μm was defined as the severest degree of SM (Foin et al., 2014). This may be due to differences between stents of various diameters and straight stents. The stents used in the experiments only had a larger detachment distance at the proximal end, while the stent in our simulation had a longer area with large detachment distances; thus, the struts near the wall did not restrict the downstream blood flow. This indicates that the detachment distance and also the length of malapposed stents together influence the amount of thrombus on a certain cross-section. When the stents feature a similar length of SM segment, the larger the detachment distance, the greater the accumulation of thrombi downstream to the distal end of the stent.

Differences in the *in vitro* environment are partly responsible for the observed error between the simulation results and *in vitro* results. The perfusion was carried out without the formation of coagulators on the tube and heparin was administered in the device to avoid coagulation; moreover, the running velocity was lower than the average blood velocity in a cardiac cycle. The focus of this study was to investigate the effect of the local hemodynamic environment surrounding malapposed stents on ST. To some extent, the experimental conditions still provide a relatively realistic environment to support our simulation results. Our study provides an idealized model that could eliminate some irrelevant factors such as stenosis vessel or curved stents, which, in addition to SM distances, would influence the blood flow.

The present study has some limitations; for example, we considered the effect both of high shear and low shear on thrombus formation, but the proportion of these two parameters within a clinical environment is unclear. In future work, we will investigate and validate the proportion of the adverse shear impact on thrombus formation to accurately simulate ST. A further limitation is that, based on the idealized model with a single variable, the model does not adequately reflect the realistic and various physiological *in vivo* environments, while the factors affecting thrombosis include differences in individual blood components and vascular morphology as well as postoperative stent conditions.

## 5 Conclusion

To understand the biomechanical association between SM and the development of thrombus, our study integrated the thrombosis calculation method into SM models. Under the shear-induced thrombus aggregation mechanism, we simulated the transport and activation of blood substances and dynamic thrombus formation through a 3D continuous model with different SM distances to investigate the quantitative association between the severity of SM and the degree of thrombus formation. The framework of thrombus growth took into account the updated fluid domain after thrombus formation and this made it possible to involve not only the effect of disturbed flow on thrombus induced by SM but also their interaction. The results revealed that the thrombogenicity of malapposed stents increased with the growing degree of SM overall but a slight drop in thrombus volume appeared at the intermediate SM (0.225 mm). Because the thrombus tended to accumulate to the distal end as the detachment distance increased, the thrombus located on the stented area did not grow larger despite the increasing thrombogenicity. Our study provides further knowledge by comparing our findings with previous *in vitro* studies to show that SM affects ST differently depending on the severity of SM and also on multiple factors related to flow velocity, stent thickness, and the length of the malapposed segment.

## Data Availability

The original contributions presented in the study are included in the article/supplementary material; further inquiries can be directed to the corresponding author.
